# Studying heating effects on desi ghee obtained from buffalo milk using fluorescence spectroscopy

**DOI:** 10.1371/journal.pone.0197340

**Published:** 2018-05-11

**Authors:** Naveed Ahmad, M. Saleem

**Affiliations:** 1 Agri. & Biophotonics Division, National Institute of Lasers and Optronics (NILOP), Islamabad, Pakistan; 2 Department of Physics, Mirpur University of Science and Technology (MUST) Mirpur, Azad Kashmir, Pakistan; Aligarh Muslim University, INDIA

## Abstract

Characterisation and thermal deterioration of desi ghee obtained from buffalo milk is presented for the first time using the potential of Fluorescence spectroscopy. The emission bands in non-heated desi ghee centred at 375 nm is labelled for vitamin D, 390 nm for vitamin K, 440–460 nm for isomers of conjugated linoleic acid (CLA), 490 nm for vitamin A and the region 620–700 nm is assigned to chlorophyll contents. Fluorescence emission spectra from all the heated and non-heated ghee samples were recorded using excitation wavelengths at 280, and 410 nm which were found best for getting maximum spectral signatures. Heating of desi ghee affects its molecular composition, however, the temperature range from 140 to 170°C may be defined safe for cooking /frying where it does not lose much of its molecular composition. Further, the rise in temperature induces prominent spectral variations which confirm the deterioration of valuable vitamins, isomers of CLA and chlorophyll contents. Fluorescence emission peak at 552 nm shows oxidation product and an increase in its intensity with the rise in temperature is observed. In order to classify heated samples at different temperatures, principal component analysis (PCA) has been applied on heated and non-heated ghee samples that further elucidated the temperature effects.

## Introduction

Desi ghee is a type of liquid butter obtained from cow/buffalo milk by adding yogurt culture[1]. It has been widely used in subcontinent (Pakistan, India and Bangladesh) and across the world at large from centuries and a best source of lipid nutrients, fat soluble vitamins and essential fatty acids[2]. Composition of desi ghee mainly consists of 62% monounsaturated fats and is rich in conjugated linoleic acid (CLA) and vitamin A[3,4]. CLA is a blend of linoleic acid isomers which accounts for 75–90% of total CLA isomers[3,5]. Due to enriched content of CLA, desi ghee has been reported as an antioxidant, anticarcinogenic, antidiabetic, antiatherogenic, and antiadipogenic properties[3]. It has also been reported for anticancer characteristics and for the prevention of cardiovascular diseases, strengthening the immune function and modifying body composition to treat obesity or build lean body mass[6,7].

Cooking and frying of food in oil or ghee is common to human and preservation of their natural ingredients during frying/cooking of food is an important issue. The molecular structures of vitamins, antioxidants, saturated and unsaturated fats in edible oils and desi ghee are very sensitive to temperature and it causes the degradation of above said nutritional ingredients. Therefore, it becomes very necessary to investigate temperature effects on molecular composition of desi ghee.

Fluorescence spectroscopy has proved itself as an excellent nondestructive analytical technique with high specificity and sensitivity for the characterization of edible oils, thermal effects on olive oil during heating and storage, thermal processing of milk fats and dry milk storage effects[8–17]. A number of research papers have been published for the investigation of temperature effects on edible oils using Fluorescence spectroscopy[11,15–20] but no study has been made so far reporting such effects on the molecular composition of desi ghee. Cheikhousman et al. employed Fluorescence spectroscopy to investigate the thermal deterioration of olive oil by heating the samples at 170°C and reported possible deterioration of different ingredients including vitamin E[16]. Microwave and conventional heating effects have been studied by applying fluorescence spectroscopy on mixtures of olive oil samples[18]. It has also been used on heated samples of olive oil mixed with sunflower oil for their discrimination[15]. F.G. Vila reported detailed molecular composition of olive oil and the effects of temperature using fluorescence spectroscopy[11]. Similarly, evolution of fluorescence compounds has been studied by fluorescence spectroscopy during the thermal deterioration of olive oil[17]. Temperature effects on extra virgin olive oil (EVOO) have been suggested a temperature range from 140 to 190°C where minimum variations in its molecular composition have been observed[19]. Literature review indicates that only a few article appear regarding desi ghee attributes[1,2,21], where gas chromatography has been employed to investigate the effects of storage on chemical properties of desi ghee[21]. In another study explained molecular composition of desi ghee along with margarines and different edible oils[22]. Recently, Saleem et al. defined a safe cooking temperature range for EVOO where it almost retains its nutritional values[23].

In the present study, Fluorescence spectroscopy has been utilized as a tool to investigate molecular composition of desi ghee obtained from buffalo milk and heating effects on its natural ingredients. In addition, fluorescence spectra of vitamins A, E, D, K and CLA are recorded and compared with the fluorescence spectra of buffalo ghee. Results suggested a temperature range that can be used for cooking/frying with desi ghee without destroying its valuable ingredients.

## Materials and methods

Buffalo ghee is very common and popular in subcontinent for frying and cooking food. In order to investigate the heating effects on its molecular composition, present study has been planned and it got approval from the ethics committee at National Institute of Lasers and Optronics Islamabad.

### Sample preparation

There are four methods commonly used for producing desi ghee: direct cream method, pre-stratification, cream butter method and milk butter/desi method. Desi method or milk butter method[1] have been adopted for the preparation of desi ghee (a local name of the clarified butter in Pakistan and India) which was extracted from the butter obtained from the buffalo milk by adding yogurt culture. By adopting desi method, fresh buffalo milk samples were obtained at the time of lactation from the local dairy farm situated in country side of Mian Channu (Punjab, Pakistan). These milk samples were boiled and cooled to the room temperature. Yogurt culture was added and left the milk to incubate from 08 to 12 hours at room temperature. After incubation time milk changed in to *Dahi* (curdled whole milk). Some water was added in *Dahi* and after churning it, desi butter with butter milk was produced in a clean metal vessel[1,2]. Obtained desi butter was heated slowly at low temperature in an open pan under continuous stirring to allow evaporation of the water without charring the proteins. At a temperature of around 110°C, desi ghee with a very pleasant fragrance and light greenish hue got fully separated from the rest of the ingredients of the butter and it took around 15 minutes. The temperature used for separation of desi ghee from cream or butter is reported from 105 to 120°C[1,2]. There are several traditional methods to extract desi ghee in different parts of the world. In Egypt, 2% salt is added in the butter while heating under continuous stirring and a handful of maize or other cereal flour is added in ghee made in Ethiopia[2]. Similarly, in our study, 1% of wheat flour was added in the melted butter at about 100°C in order to separate ghee from residue solids present in butter material. It is an ancient practice in subcontinent to add flour in heated butter which helps caramelise nonfat butter solids. The quantity of flour added was very small which does not have any effect on the molecular composition of desi ghee due to increased difference in density between fat and non-fat phases. The ghee was filtered and stored at low temperature for further analysis. Temperature variations were monitored by a K type thermocouple which measure temperature with an uncertainty of ±2°C.

In order to make heated sample of buffalo ghee, an experiment was performed in which an egg was fried with desi ghee in a frying pan with a controlled heat supply. Gas burner was used for the heat supply and the flame was kept at low value so as to achieve a possible constant heat supply to the frying pan and to avoid rapid temperature changes. The frying pan was moved up and down slowly in an effort to maintain the required temperature. The purpose of this experiment was to monitor the time and temperature taken by an egg to get fried. 100 grams of buffalo ghee was heated in a frying pan up to a temperature of 140°C and an egg was poured in pan at this temperature. Before pouring egg in frying pan, heated ghee of quantity 3 ml was sampled for acquiring its fluorescence spectra. Immediately, after pouring the egg, the temperature of ghee dropped down to around 120°C and it took around one minute to rise back to 140°C and then continued towards 150°C. A temperature ranges from 140–150°C was maintained to get the egg fully fried and whole process took around five minutes. After five minutes frying at this temperature range, egg got fully fried and its corners turned light brown and at 150°C heated ghee of quantity 3 ml was sampled again for acquiring fluorescence spectra. Further heating of ghee with egg in frying pan causes the egg to turn dark brown from light brown around temperature 180°C and then black around temperature of 200°C. It shows that food cooked at this temperature and above loses its nutritional values and got oxidized.

For detail study, separate samples of 100 grams each of desi ghee were heated in frying pan without any cooking material with same conditions and temperatures at 140, 150, 160, 170, 180, 200 and 250°C for a time period of five minutes for acquiring their fluorescence spectra. At 250°C, desi ghee started to smoke and is labelled as its smoke point. In order to confirm the fluorescence bands of ghee samples, the fluorescence spectra of conjugated linoleic acid (CLA) (Nature’s Bounty, Inc. Bohemia NY 11716 U.S.A.), vitamin K (Munawar Pharma Lahore, Pakistan), vitamin D (SAMI Pharmaceuticals Ltd.) and vitamin A (Matrix Pharma Karachi, Pakistan), vitamin E (Tocopherol (Evion capsules) Merck pharma, Pakistan) were purchased from the local market of Islamabad, Pakistan and their spectra were recorded and compared.

### Acquiring of fluorescence spectra

**Fig 1:**
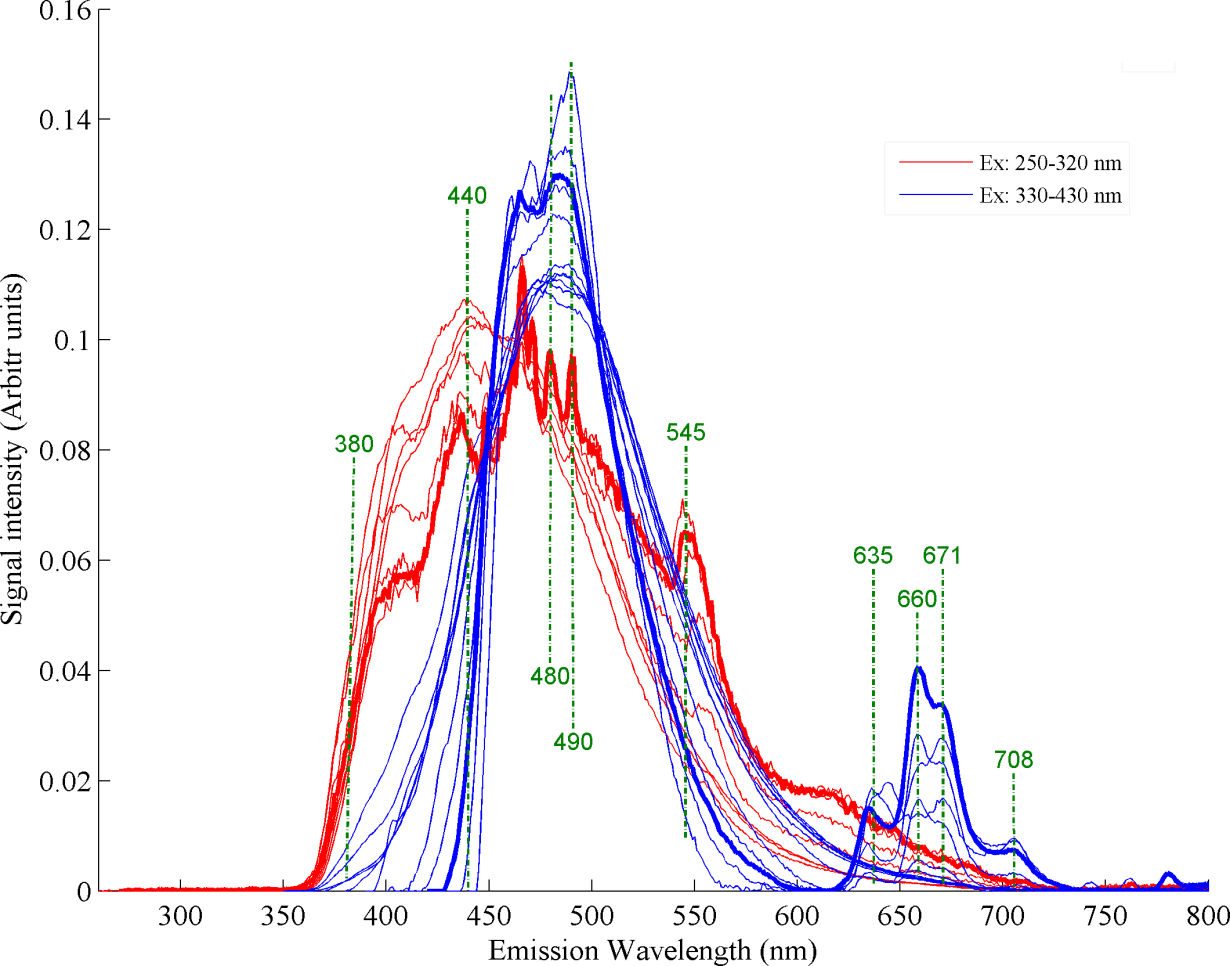
Fluorescence spectra of non-heated buffalo ghee at excitations wavelengths from 250–430 nm.

In this study, a spectrofluorometer (FluoroMax-4, Horiba scientific, Jobin Yvon, USA) with xenon light source has been employed to record the fluorescence spectra from all heated and non-heated desi ghee samples. Buffalo ghee samples in molten form were poured in a square cuvette and the emission spectra were recorded. In order to record closely spaced emission spectra, width of slits was fixed at 3 nm for excitation and 2 nm for emission monochromators. A fresh non-heated desi ghee sample was excited with different wavelengths from 250 to 430 nm with steps of 10 nm. The excitation bands from 250–320 nm and 330–430 nm result in emission spectra of similar shape of corresponding range, are shown in Fig 1. Among these excitation wavelengths, two excitation wavelengths at 280 and 410 nm with maximum spectral information were selected to record the emission spectra of the ghee samples which were heated at temperatures of 140, 150, 160, 170, 180, 200 and 250°C. For each excitation wavelength, 10 emission spectra were recorded from each sample to eliminate any instrumental and human error. Similarly, fluorescence spectra from the samples of vitamins A, D, E, K and CLA were recorded using excitation wavelengths of 280 and 410 nm. For vitamins and CLA, three spectra for each were recorded and their average was plotted for each wavelength to eradicate any instrumental or human error.

### Preprocessing of fluorescence spectra

All the fluorescence spectra of ghee samples were pre-processed before analysis using Savitzky-Golay smoothing method (order 5, 13-point window) in MATLAB (Mathworks, release 2014) environment through a self-written code. This has been employed to remove random noise evolving during recording the spectra. After smoothing, the emission spectra highlighted peaks confirming the presence of different molecular structures in the buffalo ghee. In addition, all emission spectra were vector normalization for the better comparison and analysis of different molecular bands. Further, classification of small spectral variations induced as a result of heating was obtained by applying principal component analysis (PCA), a widely used statistical tool through a MatLab built-in routine. As a result of PCA, resulting classification in the heated and non-heated ghee samples are explained by scatter plots between first two principal components PC1 and PC2 in the relevant sections.

## Results and discussions

### Spectral description of non-heated buffalo ghee

Fluorescence spectra of non-heated buffalo ghee recorded with different excited wavelengths from 250–430 nm is displayed in Fig 1. Analysis of ghee spectra evidently suggests the selection of excitation wavelengths at 280 and 410 nm where they fluoresce maximum spectral information for studying the heating effects on its molecular composition. Fig 1 shows prominent fluorescence bands at 380, 440, 490, 552, 635, 660, 671 and 708 nm. The fluorescence band at 380 nm in buffalo ghee accounts for fat soluble vitamins and also shows traces of carotenoids and this band also appears in buffalo milk so verifying the presence of these ingredients [24–26]. Bands at 440 and 490 nm are associated to isomers of CLA and vitamin A respectively. Fluorescence band at 552 nm is assigned to oxidation product which evolved due to heating during extraction process. The region from 620–700 nm can be assigned to chlorophyll[11,12,17] contents.

**Fig 2:**
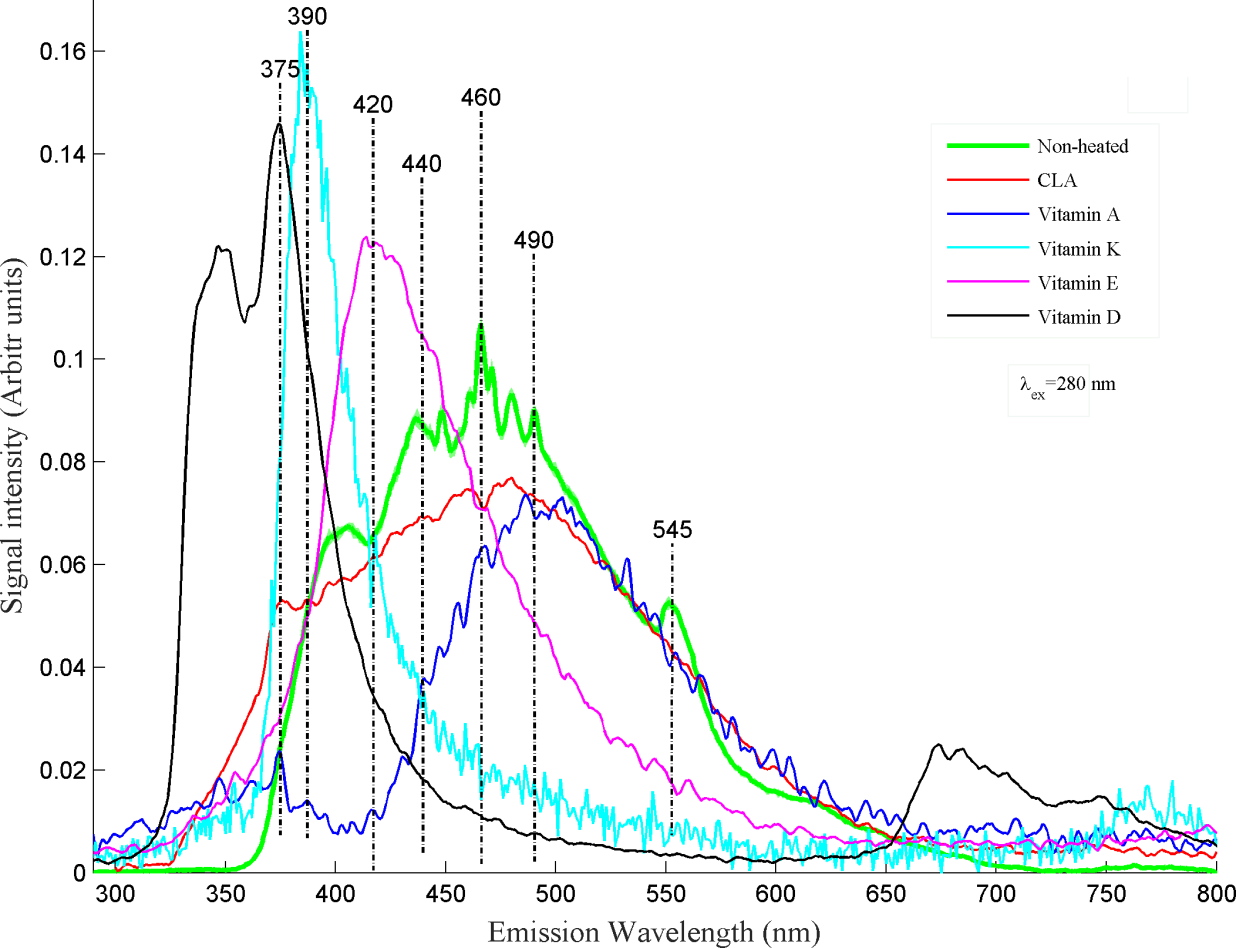
It shows comparison between fluorescence spectra of buffalo ghee and vitamin A, D, E, K and CLA for excitation wavelength of 280 nm.

For the comparison of assignments of fluorescence bands in Fig 1, fluorescence spectra of non-heated desi ghee along with spectra of vitamins A, D, E, K and CLA obtained at excitation wavelengths of 280 nm is shown in Fig 2. CLA is reported for the prevention of cardiovascular diseases, anticancer attributes, help strengthen the body immune system and general human health[6,7]. It also helpful in reducing adiposity in human[27] which is a very common problem across the world. Desi ghee obtained from buffalo milk has high concentration of CLA which acts as an antioxidant[3]. Fluorescence spectra of CLA at λ_ex_: 280 nm gives a broad band emission spectra form 350–600 nm centered roughly at 460 nm, which may be assigned for Isomers of CLA. Spectra of CLA completely overlaps with the desi ghee spectra giving rise to the fact that CLA isomers are widely present in buffalo ghee which is in accordance with literature[3].

According to World Health Organisation (WHO) about 45% of vitamin A deficient and xerophthalmic women and children habitat in south and southeast Asia[28]. Pakistan is also facing many health problems including deficiency of vitamin A which lead to the effects like anemia, eye retinal disparity and diarrhea[29]. Fluorescence spectrum of Vitamin-A shows its strong overlapping with spectra obtained from buffalo ghee which is obvious from the fact that buffalo milk is rich with vitamin A[4]. Its concentration in the buffalo milk has been reported as 340 IU.mL^-1^[30]. Therefore, an adequate quantity of buffalo ghee in daily food can be helpful to eradicate deficiency of this vitamin.

Vitamin E is one of the most important lipid soluble nutrients and its severe deficiency can cause cystic fibrosis, chronic cholestatic liver disease, abetalipoproteinemia, short bowl syndrome and can have severe effects on central nervous system of human body[31]. Vitamin E acts as an antioxidant and help maintain cell bioactivity in human body[32,33]. Fig 2 shows vitamin E spectra in the form of a broad band from 350–600 nm covering almost half of desi ghee spectra which confirm its presence in buffalo ghee as buffalo milk fat contains high level of natural antioxidant tocopherol(vitamin E)[30]. Daily use of buffalo ghee can provide human with the requirements of vitamin E and may prevent them from several diseases.

The emission spectrum of vitamin K cantered at 385 nm covers the shoulder of buffalo ghee spectrum. Vitamin K help stop bleeding by rapid coagulation of blood[34] and its deficiency increases the risk of bone fracture[35]. Therefore, its adequate quantity in daily food becomes vital for human health and ghee is one of the good sources available for this vitamin.

Fig 2 shows fluorescence spectra of vitamin D, where a prominent emission band appears at 375 nm when excited at 280 nm and shows its presence in buffalo ghee. The deficiency of vitamin D can cause cardiovascular diseases and rickets[36,37].Vitamin D can give protection against cancer, diabetes, heart disease and osteoporosis[38] and thus plays an important role to help maintain human health. Therefore, buffalo ghee can be used to meet the requirement of vitamin D for human body.

### Effects of heating on desi ghee

Heating introduces prominent effects on the natural molecular composition of desi ghee due to thermal oxidation of fats, vitamins and CLA isomers. Desi ghee retains its molecular composition to a temperature range which is investigated in this study by fluorescence spectra which are recorded by using excitation wavelengths 280 and 410 nm as explained in the following paragraphs.

#### Studying heating effects at λ_ex_: 280 nm

**Fig 3:**
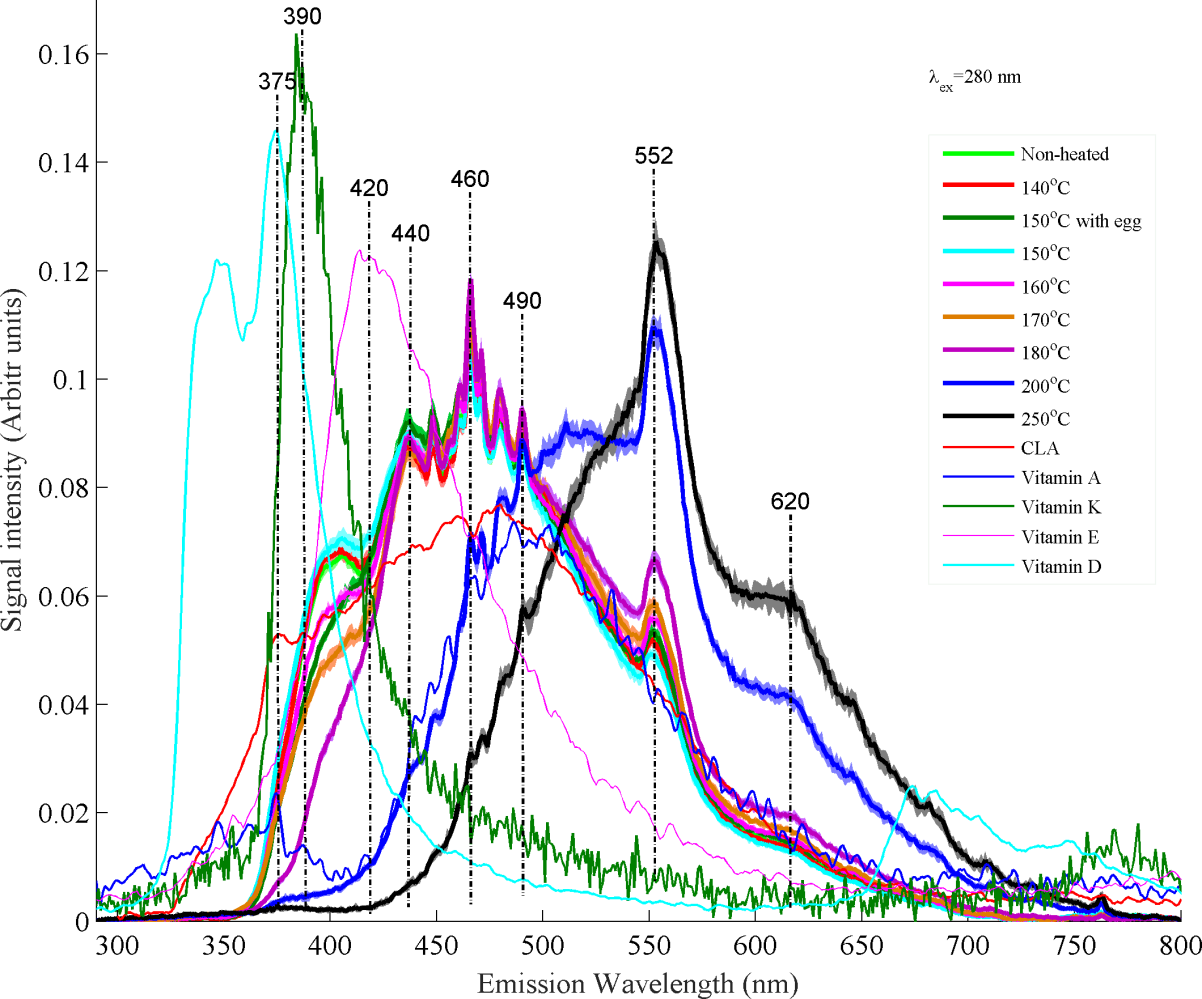
Fluorescence spectra obtained at excitation wavelength of 280 nm show the comparison between non-heated ghee samples, heated ghee samples, CLA and vitamins A, D, E and K.

Fig 3 shows spectra of non-heated and heated samples of desi ghee excited at λ_ex_: 280 nm. In this figure, standard deviation is plotted at each fluorescence spectrum of heated samples which appears as shaded area and its small value confirms the repeatability of data. The samples were heated at a temperature of 140, 150 with egg, 150 without egg, 160, 170, 180, 200 and 250°C for a time period of 5 minutes for each sample. The purpose of this experiment was to find possible changes in fluorescence spectra of desi ghee heated at different temperatures for 5 minutes during which cooking/frying of food is usually carried out but it also depends upon the quantity of the material and heating sources.

It is clear from Fig 3 that vitamins A, E and CLA contents apparently present from 380–500 nm sustained with heating at temperature of 140–150°C and started to deteriorate for further heating as a result of thermal oxidation. The spectral variations in the molecular composition of desi ghee heated at 150°C with egg and without egg are almost same which explains that the presence of egg for frying does not affect its molecular composition to a large extent. At temperatures above 150°C band at 552 nm which is assigned to oxidative products[11] shows a gradual upward increase in its intensity with an increase in temperature starting from 160°C and becomes maximum at 250°C. Similarly, fluorescence band at 620 nm may also be assigned to oxidative products because its trend of intensity rise is similar as observed in case of fluorescence emission band at 552 nm however, it is not reported in literature. Actually, as temperature goes above 150°C primary oxidation products produced which are unstable and immediately decomposes to secondary oxidation products with the rise in temperature like alcohols, saturated & unsaturated aldehydes and epoxy compounds are reported[39]. In case of desi ghee heating in present study, possibly similar type of compounds appear with heating as temperature goes above 150°C, primary oxidation products started to appear with heating and decomposes to secondary oxidation products at 180–200°C with prominent spectral variations indicating severe damage to the natural ingredients of desi ghee. When temperatures increase from 200–250°C final oxidation products such as aldehydes and ketones produced with a rancid smell form the desi ghee and spectra at this temperature appear to be different from all the rest. Fluorescence bands at 390, 440, 460 and 490 nm show a gradual decrease in intensity as temperature goes above 150°C indicating the thermal oxidation of monounsaturated fatty acids, vitamins and CLA isomers. Also, In the first region of Fig 3 from 350–490 nm, an overall decrease in intensity of the peaks can be observed which can be associated to the oxidation of fat soluble vitamins K, D and E and while in the later region from 490–650 nm an overall increase in the intensity can be observed which can be associated to the oxidation of vitamin-A isomers with the vital evolution of oxidative products at 552 and 620 nm. Isomers of CLA present in large concentration shows deterioration in both regions with the rise in temperature.

**Fig 4:**
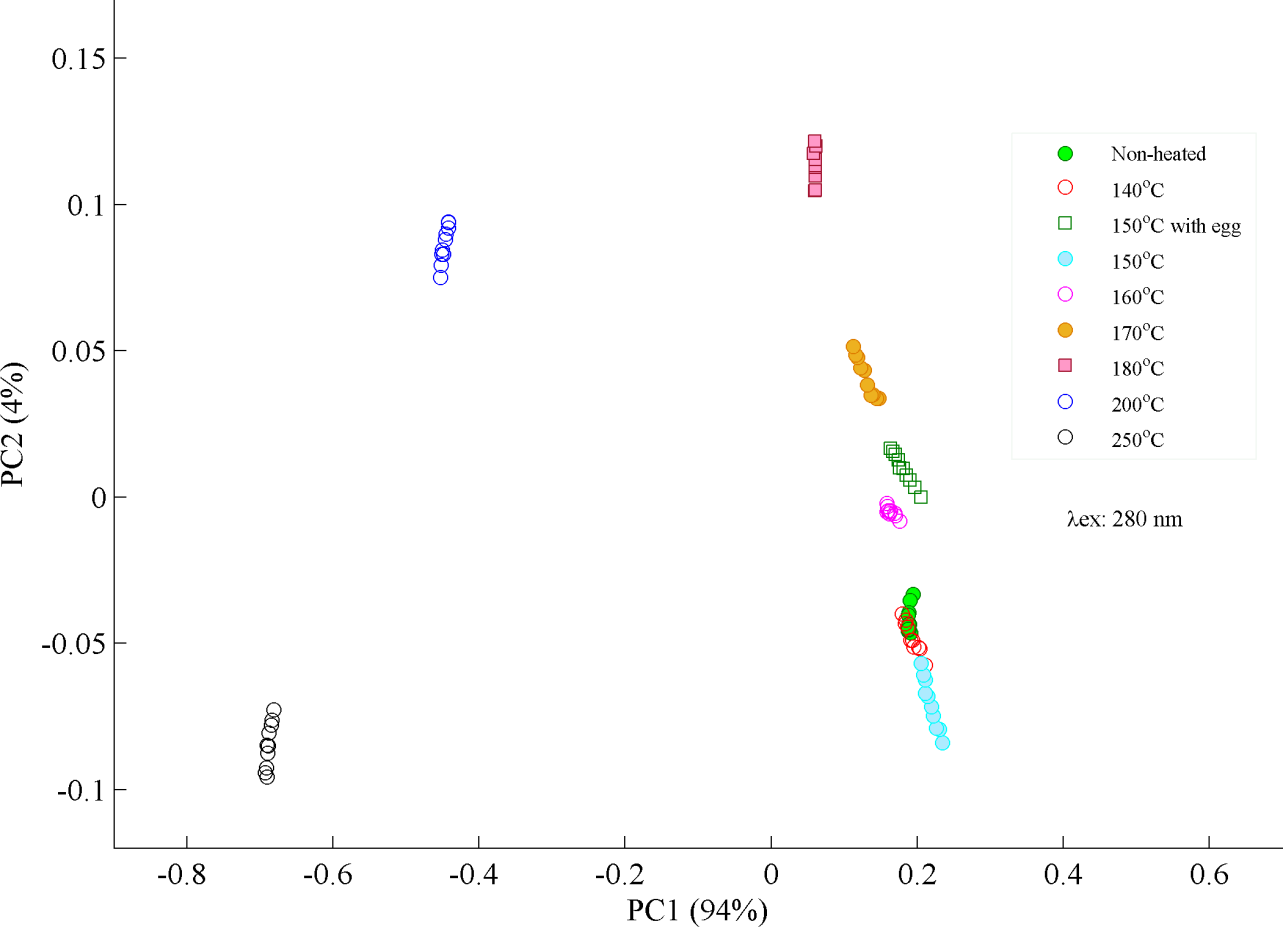
PCA scatter plot is displayed between PC1 and PC2 which classifies heated and non-heated ghee samples based on their spectral variations excited at 280 nm.

Principal component analysis (PCA) has been employed to classify different overlapping spectral signatures which are hard to observe by naked eye. PCA is a non-supervised statistical procedure which uses an orthogonal transformation to convert a set of observations of possibly correlated variables into a set of values of uncorrelated variables named principal components. These principle components are not correlated and are orthogonal to one another. These principal components are calculated based on the variance in the data and when plotted as a scatter plot, they nicely separate different set of data. First two or three components usually give maximum information about the variance in the data[40] sets and if they are completely orthogonal to each other, then their score plot between PC1 and PC2 separate both components. In PCA score plot shown in Fig 4, heated samples from 140–150°C are not separable from the non-heated one which show stability of buffalo ghee for said temperature range for a heating time period of 5 minutes. Ghee samples heated at 150°C with egg clustered a little away from samples heated at 150°C without egg near samples heated at 160°C which explains the possible oxidation of ghee samples due to egg contamination. But it does not show a very strange behavior and lies near the samples heated from 140–150°C without egg. Even samples heated at 160 and 170°C are lying close to non-heated or heated from 140–150°C samples. In contrast, samples heated at 180°C and above clustered far from the non-heated or heated from 140–150°C and can be observed in the fluorescence spectra in Fig 3. Desi ghee samples heated at 200 and 250°C clustered on negative side of the PCA score plot showing very different behavior from the rest and also appear very different in Fig 3. Therefore, the temperature ranges from 140–170°C may be considered as safe for frying/cooking in order to preserve molecular composition of desi ghee during cooking.

**Fig 5:**
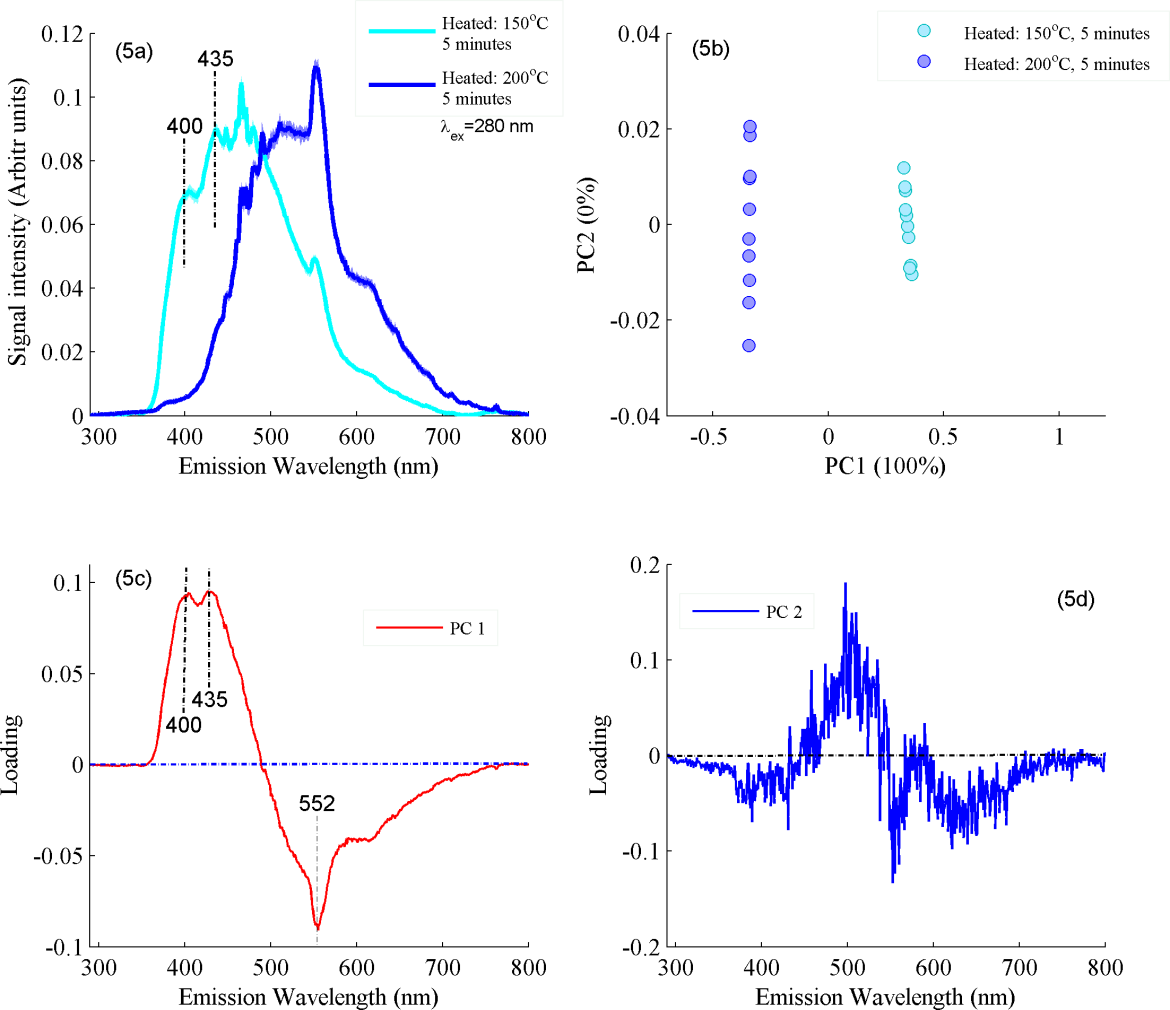
(a) Fluorescence spectra between ghee samples heated at 150 and 200°C at excitation wavelength of 280 nm. (b) PCA scatter plot is displayed between PC1 and PC2 for the classification of samples heated at 150 and 200°C. (c) Loading vector of first principle component PC1. (d) Loading vector of second principle component PC2.

In order to illustrate that PCA classification is based upon its loading vectors, two samples heated at 150 and 200°C were selected with prominent spectral differences to produce their PCA scatter plot between PC1 & PC2 are shown in Fig 5A and 5B and their loading vectors in Fig 5C and 5D. Fig 5A shows Fluorescence spectra of ghee samples heated at 150 and 200°C and evidently explains that Fluorescence emission bands at 400, 435 and 452 nm show variations in intensity/shapes of bands which are induced as a result of heating. Fig 5B illustrated that the samples heated at 150°C are clustered in the positive side and samples heated at 200°C in the negative side of the PC1 axis which follows from the spectral features associated with the former are loaded positively, while those of the later are loaded negatively[41]. Fig 5C shows PC1 loadings and corresponding labelling of bands. It is clear from PC1 loadings that Fluorescence emission bands at 400 and 435 nm are loaded positively while at 552 nm are loaded negatively which shows that heating at 200 induces prominent changes in these molecules as compared to heating at 150°C. Therefore, it can be concluded that PC1 mainly separates the two-data set with maximum variance of 100% while PC2 gives only noise. This shows that PC1 and PC2 are independent of each other which causes the separation of both types of data when score plot is produced. It means that features identified in Fig 4 are also feature in the loadings of PC1.

#### Studying heating effects at λ_ex_: 410 nm

**Fig 6:**
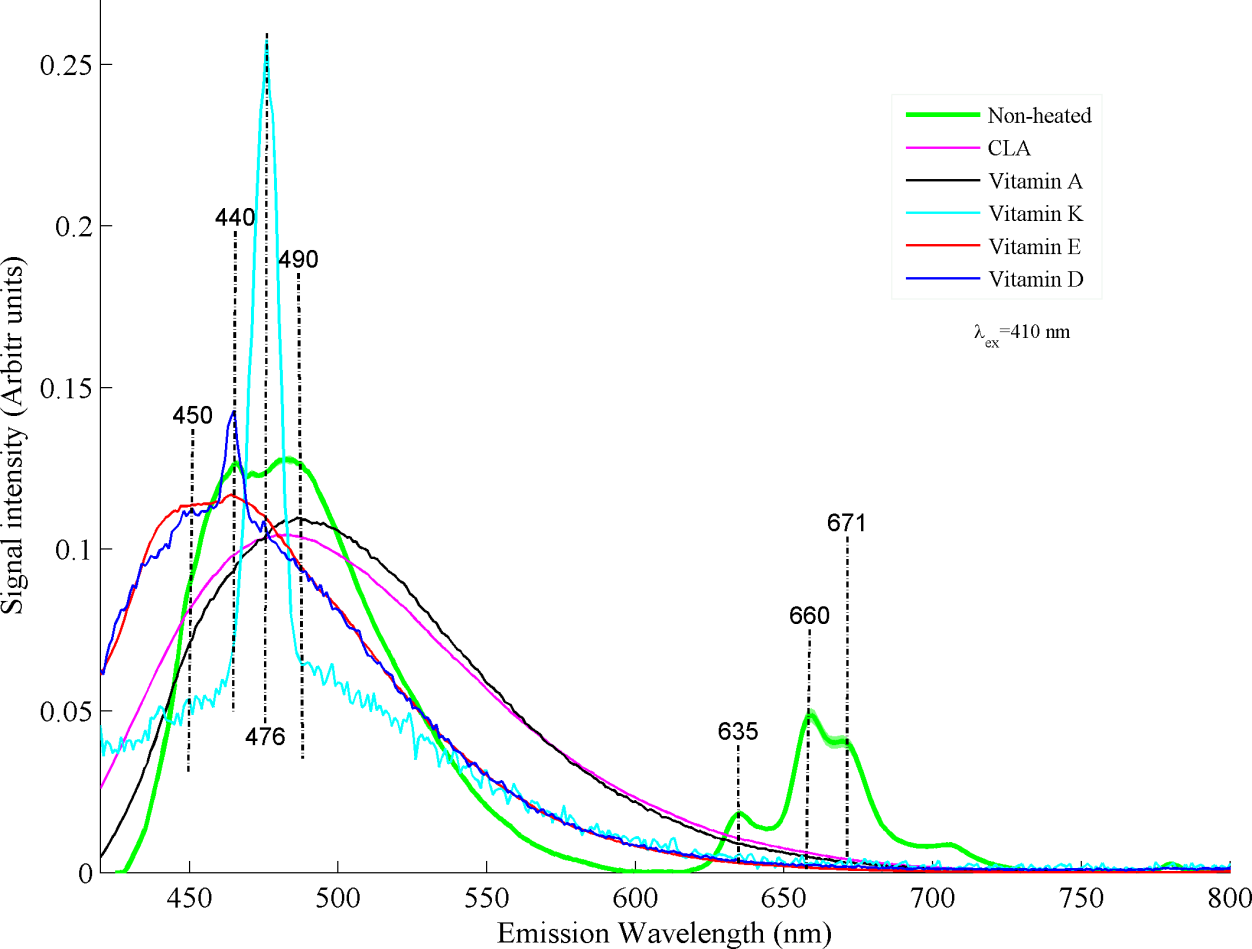
It shows comparison between fluorescence spectra of buffalo ghee and vitamin A, D, E, K and CLA for excitation wavelength of 410 nm.

Fig 6 shows a nice comparison between fluorescence emission spectra of non-heated buffalo ghee with vitamin A, D, E, K and CLA excited at λ_ex_: 410 nm. Fluorescence emission band positions of non-heated desi ghee and vitamins are different from emissions excited at 280 nm. This shift/change in emission bands possibly explain the wavelength dependence behaviour of fluorophores present in buffalo ghee under different excitation wavelengths. With excitation wavelength of 410 nm, non-heated buffalo ghee shows prominent fluorescence bands at 450, 460, 476, 490, 635, 660 and 671 nm. These emission spectra can be divided into two regions: region one can be assigned to emission band from 430–560 nm centered roughly at 490 nm is assigned to vitamin-A, bands at 450 and 460 nm are assigned to vitamin E and D, band at 476 assigned to Vitamin-K contents and band at 490 nm is assigned to vitamin A respectively. In second region from 620–700 nm, the peaks at 635, 660, 671 and 700 nm are assigned to chlorophyll contents.

**Fig 7:**
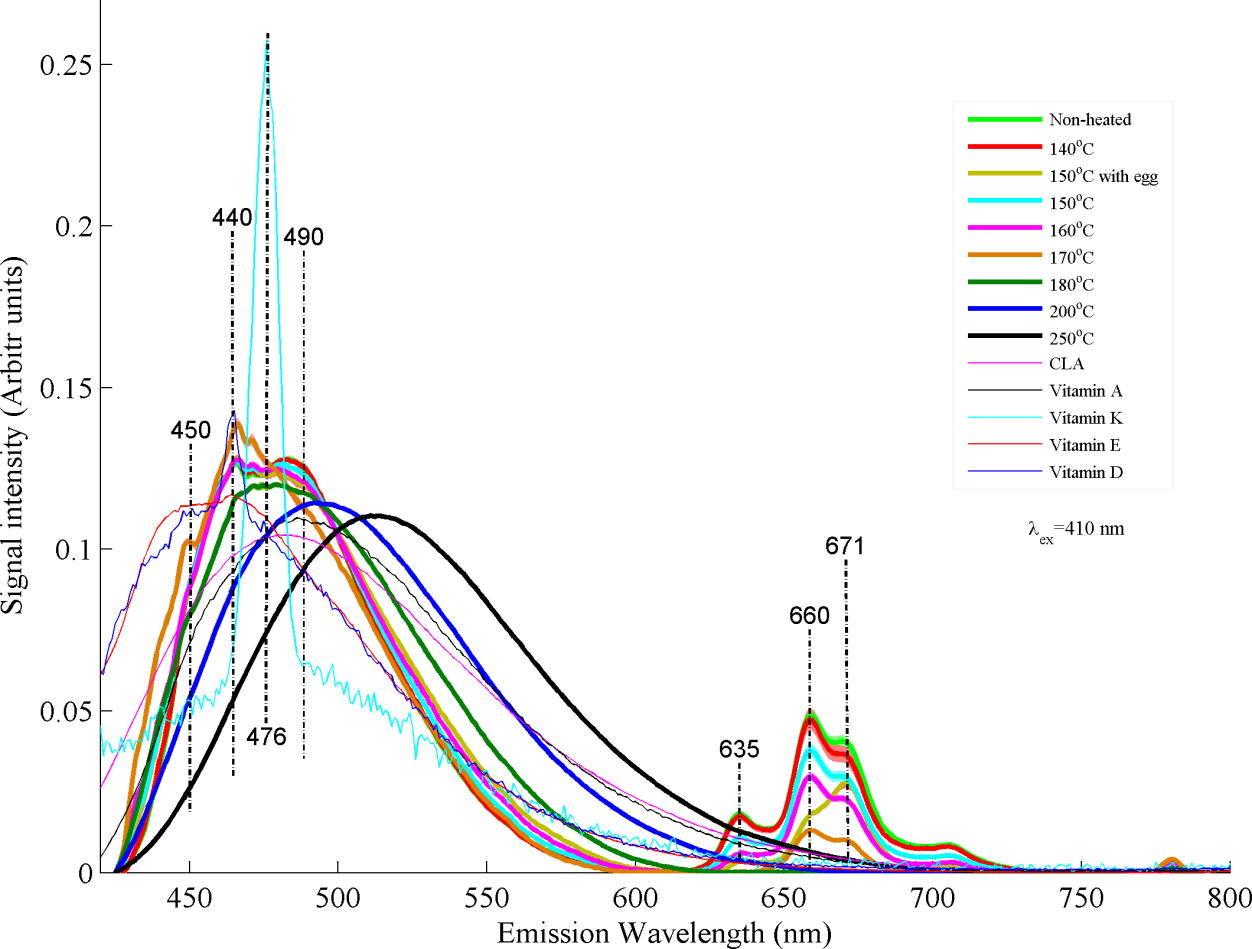
Fluorescence spectra obtained at excitation wavelength of 410 nm showing comparison between non-heated ghee samples, heated ghee samples, CLA, vitamins A, D, E and K.

Fig 7 shows comparison of fluorescence spectra of non-heated and heated desi ghee samples with all the vitamins and CLA. Desi ghee samples were heated from 140–250°C along with ghee samples heated at 150°C with egg for a time period of 5 minutes for each sample. It can be observed that samples heated at 140, 150°C without egg, 150°C with egg and 160°C are not separable apparently from non-heated samples in the first region from 430–550 nm. This observation leads to the fact that desi ghee retains its vitamins and CLA composition up to a temperature of 160°C for a heating time of 5 minutes. In second region 620–700 nm samples hated at 140°C are not separable from non-heated ones. Samples heated at 150°C with egg shows more oxidation of chlorophyll as compared to samples heated at 150°C without egg and 160°C which appear mainly in the form of change in color of desi ghee. This increase in oxidation corresponds to the contamination induced by egg during frying process. Samples heated at 170°C shows rise in intensity in first region and decrease in the second region. An appreciable spectral change occurs for the samples heated at 180°C with a shift in whole band towards higher wavelength values in the first region and a complete oxidation of chlorophyll contents in the second region. The natural ingredients undergo thermal oxidation which fluoresce in the form of broad bands and shifted towards higher wavelength values. In fact, valuable ingredients of buffalo ghee start to deteriorate as temperature rise above 150°C as a result of thermal oxidation with the initiation of primary to secondary oxidation products and finally end at final products like ketones and aldehydes at smoke temperature[39]. Spectral changes at 180°C possibly explain the evolution of secondary oxidative products and this trend continues for the temperatures of 200 and 250°C as can be observed from Fig 7. Fluorescence spectra of buffalo ghee heated at 200°C shows a prominent shift towards higher wavelength showing maximum oxidation of valuable contents like vitamins and isomers of CLA as compared to non-heated ghee samples. Heating of desi ghee at temperature of 250°C induces further changes in the molecular composition of ghee leading to the evolution of final products like ketones and aldehydes with rancid smell. It shows a complete deterioration of natural molecular ingredients of desi ghee.

**Fig 8:**
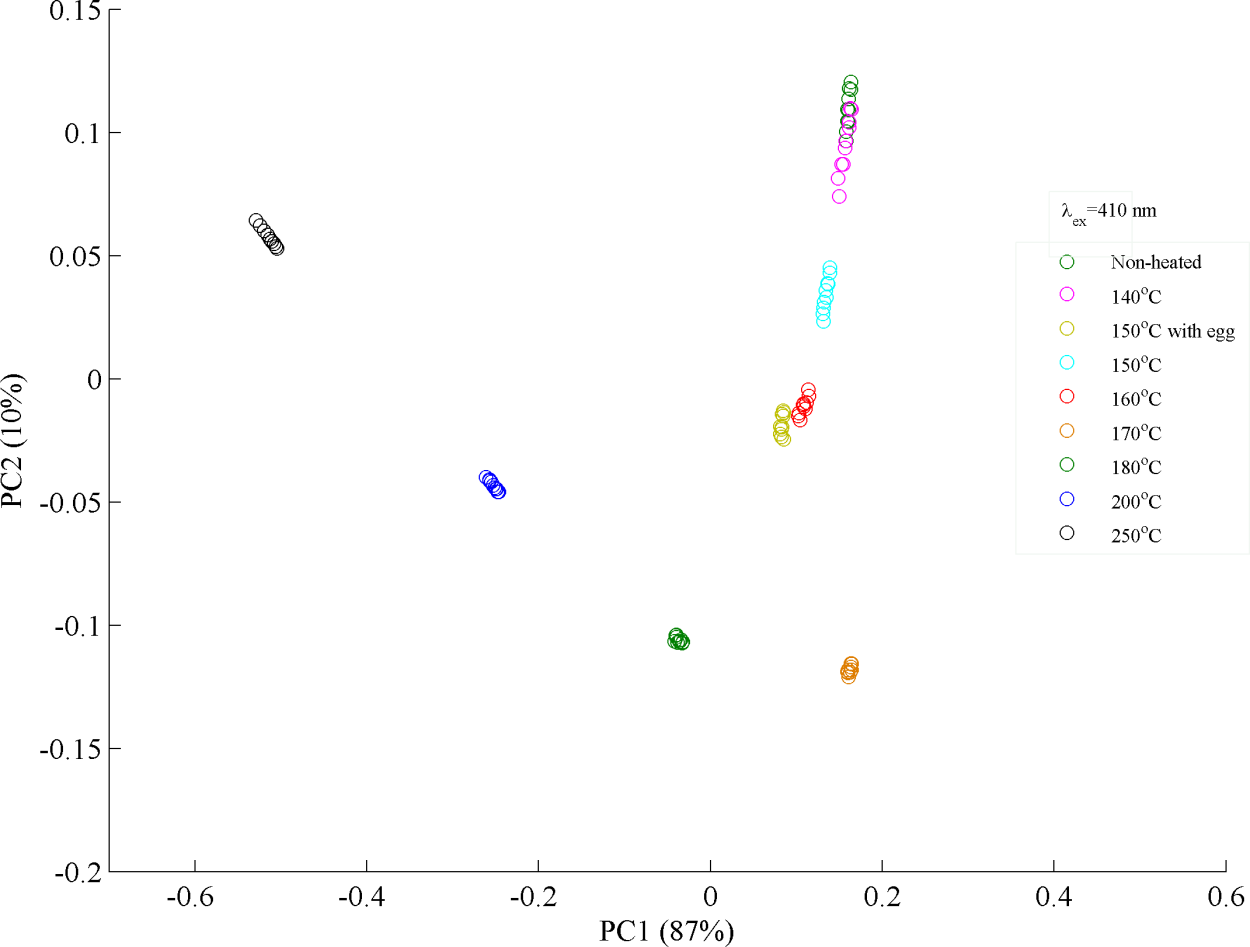
PCA scatter plot is displayed between PC1 and PC2 which classifies heated and non-heated ghee samples based on their spectral variations excited at 280 nm.

Fig 8 is a PCA scatter plot where non-heated ghee samples clustered with samples heated at 140°C. Samples heated at 150 and 160°C clustered nearer to the non-hated ones as compared to samples heated at 150°C with egg. Samples heated at 150°C with egg shows more deterioration as compared to heated at 140–160°C due to possible contamination during frying process. Ghee samples heated above 170°C shows a separating trend from the rest. Samples heated at 180, 200 and 250°C clustered away from the rest showing prominent deterioration in the natural molecular structure of desi ghee. These finding confirms the oxidation of desi ghee that started at a temperature of 170°C and induces small spectral variations up to 180°C. Buffalo ghee loses most of its natural ingredients at a temperature of 200°C and turned to rancid material like aldehydes and ketones as final products at 250°C as clearly visible in PCA plot. Therefore, temperature range from 140–170°C is defined as safe for cooking/frying purposes using desi ghee and up to 180°C with small deterioration in its natural ingredients.

**Fig 9:**
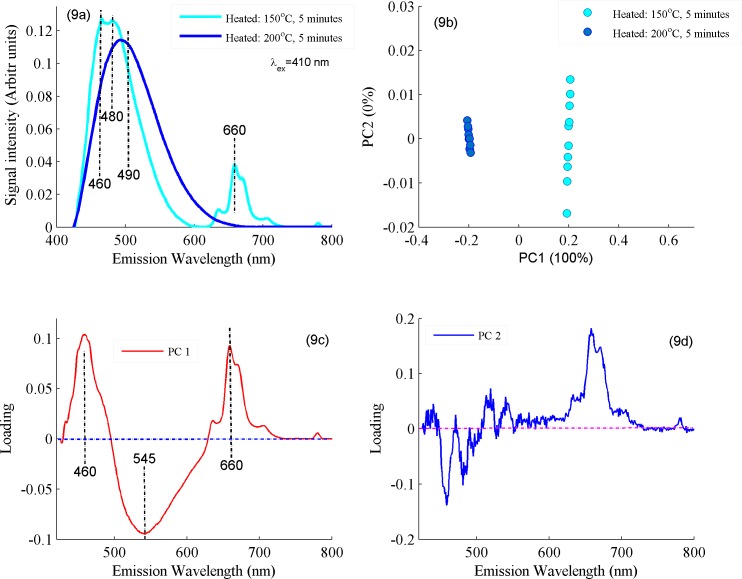
(a) Fluorescence spectra between ghee samples heated at 150 and 200°C at excitation wavelength of 410 nm. (b) PCA scatter plot is displayed between PC1 and PC2 for the classification of samples heated at 150 and 200°C. (c) Loading vector of first principle component PC1. (d) Loading vector of second principle component PC2).

For more detailed analysis, two heated samples at 150 and 200°C were selected to produce their fluorescence spectra, PCA scatter plot between PC1 & PC2 and loading vectors as shown in Fig 9A–9D. Fig 9A shows fluorescence spectra between samples heated at 150 and 200°C for a time period of 5 minutes. Fig 9B shows PCA score plot where samples heated at 150°C clustered in the positive side while samples heated at 200°C are clustered in the negative side of the PC1 axis which follows from the spectral features associated with the former are loaded positively, while those of the later are loaded negatively as shown in loadings of PC1. Evidently, positive loadings can be observed at fluorescence bands appearing at 460 and 660 nm and negative loadings at 545 nm. PC1 explains the maximum variance (100%) between two data set. Fig 9D shows PC2 as compared to PC1 where maximum variance has been explained. These features show a relative intensity change with the rise of temperature which is due to the oxidation of valuable lipid nutrients, vitamins and isomers of CLA at high temperatures of 200°C as compared to 150°C. The spectral changes associated with PC1 and PC2 are not correlated, therefore score plot between PC1 & PC2 completely separate both data set. This explanation therefore suggested that the classification of different data groups shown in Fig 8 is based on the molecular changes which appeared due to heating of ghee samples. These findings suggest that temperature range from 140 to 170°C may be used for safe frying/cooking with buffalo ghee without undergoing appreciable changes in its natural molecular structure and food ingredients also remain safe under proper heating conditions.

## Conclusion

Fluorescence spectroscopy has been employed for the characterization of buffalo ghee for the first time. It was found that desi ghee is enriched with CLA, vitamin-A and small quantities of vitamin D, E and K. In addition, emission spectral region from 620–700 nm has been assigned to chlorophyll. It was observed that up to a temperature of 150°C, desi ghee remains stable when heated for a time period of five minutes. Heating of desi ghee from 140–150°C does not affect its natural molecular composition and this temperature range can be used for household cooking/frying purposes. However, up to temperature of 170°C, ghee can be used for cooking/frying with less deterioration of valuable ingredients. Above temperature of 180°C, it deteriorates more and more and becomes rancid at 250°C.
